# Head-to-Head Comparison of the Hypoglycemic Efficacy and Safety Between Dipeptidyl Peptidase-4 Inhibitors and α-Glucosidase Inhibitors in Patients With Type 2 Diabetes Mellitus: A Meta-Analysis of Randomized Controlled Trials

**DOI:** 10.3389/fphar.2019.00777

**Published:** 2019-07-10

**Authors:** Zhaoliang Li, Liang Zhao, Leilei Yu, Jie Yang

**Affiliations:** Department of Endocrinology, the Central Hospital of Tai’an City, Tai’an, China

**Keywords:** dipeptidyl peptidase-4 inhibitors, α-glucosidase inhibitors, type 2 diabetes mellitus, meta-analysis, postprandial glucose

## Abstract

**Background:** The α-glucosidase inhibitors (AGIs) are commonly prescribed in Asian patients with type 2 diabetes mellitus (T2DM), but with a high incidence of gastrointestinal side effects. This study was aimed to compare the efficacy and safety of dipeptidyl peptidase-4 (DPP4) inhibitors and AGIs in T2DM patients in a meta-analysis.

**Methods:** Randomized controlled trials were identified *via* systematic search of PubMed, Embase, and Cochrane’s Library databases from inception to February, 2019. Meta-analyses were performed *via* a random or a fixed effect model according to the heterogeneity.

**Results:** Eighteen studies with a total of 4,051 patients with T2DM were included. The DPP4 inhibitors were associated with lower reduction of glycosylated hemoglobin (HbA1c) as compared with AGIs [weighed mean difference (WMD): −0.37%, *p* < 0.001]. Subgroup analyses indicated that the benefit of DPP4 inhibitors as compared with AGIs on HbA1c were independent of study design, scale, baseline HbA1c, with or without concurrent medications, or follow-up durations. Moreover, compared to AGIs, DPP4 inhibitors was associated with lower reductions of fasting blood glucose (WMD: −0.53 mmol/L, *P* < 0.001) and postprandial glucose at 2h (WMD: −0.60 mmol/L, *P* = 0.04), moderately increased body weight (WMD: 0.34 kg, *P* = 0.02), and decreased risk of gastrointestinal adverse events [risk ratio (RR): 0.48, *P* < 0.001], but unaffected risk of symptomatic hypoglycemia (RR: 0.96, *P* = 0.90).

**Conclusions:** The DPP4 inhibitors are superior to AGIs in T2DM patients for better glycemic control and lower risks of gastrointestinal side effects.

## Introduction

The α-glucosidase inhibitors (AGIs) are commonly prescribed oral antidiabetic drugs (OADs) for patients with type 2 diabetes mellitus (T2DM), particularly for the Asian patients (Chu et al., 2017; Gao et al., 2018; Jeon et al., 2018). In China, AGIs are recommended as the second-line therapy for T2DM if optimal glycemic control could not be achieved by the first-line therapy of metformin (Weng, 2016; Cai, 2019). Acting as inhibitors of α-glucosidase located in the brush border of the small intestine, AGIs reduce postprandial glucose (PPG) level *via* attenuating the digestion of carbohydrates, with an increased incidence of gastrointestinal side effects (Wu et al., 2017; Gao et al., 2018). The dipeptidyl peptidase-4 (DPP4) inhibitors are a novel category of OADs which inhibit the inactivation of glucagon-like peptide-1 (GLP-1) and glucose-dependent insulinotropic peptide (GIP) (Chen et al., 2015). It is suggested that DPP4 inhibitors may improve glycemic control with less risk of gastrointestinal adverse side effects compared to AGIs. However, results of previously randomized controlled trials (RCTs) were not consistent (Pan et al., 2008; Iwamoto et al., 2010a; Iwamoto et al., 2010b; Seino et al., 2011; Kawamori et al., 2012; Okada et al., 2013; Kobayashi et al., 2014; Mikada et al., 2014; Nakamura et al., 2014; Oe et al., 2015; Wang et al., 2015; Yokoh et al., 2015; Fujitani et al., 2016; Matsushima et al., 2016; Mori et al., 2016; Du et al., 2017; Koyama et al., 2018; Parthan et al., 2018). Therefore, we performed a meta-analysis for the head-to-head comparison of the hypoglycemic efficacy and safety outcomes between DPP4 inhibitors and AGIs in patients with T2DM.

## Materials and Methods

This meta-analysis was designed, performed, and presented in accordance with the Preferred Reporting Items for Systematic Reviews and Meta-Analyses (PRISMA) statement (Moher et al., 2009) and Cochrane’s Handbook guideline (Higgins and Green, 2011).

### Database Search

We performed the initial electronic database search of the PubMed, Embase, and Cochrane’s Library from inception to February, 2019, *via* the combination of the following terms: 1) “DDP4” OR “DDP-4” OR “DPP4 inhibitors” OR “sitagliptin” OR “vildagliptin” OR “linagliptin” OR “saxagliptin” OR “alogliptin” OR “dutogliptin,” 2) “alpha-GIs” OR “alpha-glucosidase inhibitors” OR “acarbose” OR “voglibose” OR “miglitol,” and 3) “random” OR “randomly” OR “randomized” OR “randomised.” The search was limited to studies in human, and no restriction was applied for the language of publication. The final literature search was performed on February 12th, 2019.

### Inclusion and Exclusion Criteria

Studies were included if they met the following criteria: 1) designed as a parallel group RCT; 2) included adult patients with T2DM; 3) allocated to an intervention group of oral DPP4 inhibitor and a control group of AGI, with or without coadministration of other oral antidiabetic medications, such as metformin and/or sulfonylureas et al.; 4) with a follow-up duration of at least 8 weeks; and 5) reported at least one of the following outcomes: changes of glycosylated hemoglobin (HbA1c), fasting blood glucose (FBG), PPG at 2h, and body weight from baseline, and the incidences of symptomatic hypoglycemia and any gastrointestinal adverse events (GIAEs) in patients of both groups. Reviews, crossover trials, preclinical studies in animals, and repeated reports of already included RCTs were excluded.

### Data Extraction and Quality Evaluation

We extracted the following study characteristics of each RCT: 1) location of the study; 2) design characteristics: single-blind, double-blind, or open label; 3) patients’ characteristics: number, age, gender, baseline HbA1c, body mass index (BMI); 4) details of background OADs; 5) regimens of DPP4 inhibitors and AGIs; and 6) follow-up durations. The quality of the included RCT was evaluated by the Cochrane’s risk of bias tool (Higgins and Green, 2011), which is based on the following seven domains: random sequence generation, allocation concealment, blinding in performance, blinding in outcome detection, incomplete outcome data, reporting bias, and the potential risk of other bias. The processes of database search, study identification, data extraction, and quality evaluation were independently performed by two authors. Discussion with a third author was indicated when discrepancies occurred.

### Statistical Analyses

The statistical analyses were performed with RevMan software (version 5.1; Cochrane Collaboration, Oxford, UK) and Stata software (version 12.0; Stata Corporation, College Station, TX). Continuous variables were analyzed using weighed mean difference (WMD) and 95% confidence interval (CI), while categorized variables were analyzed using risk ratio (RR) and 95% CI. The heterogeneity among the included RCTs was evaluated by the Cochrane’s *Q* test (Higgins and Green, 2011) and a *P* < 0.10 indicating significant heterogeneity. We also used *I* ^2^ statistic, which describes the percentage of total variation across studies that is due to heterogeneity rather than chance (Higgins et al., 2003), as an indicator of heterogeneity, and an *I* ^2^ > 50% indicates significant heterogeneity. A fixed effect model was used to pool the results of individual study during meta-analysis, if the heterogeneity was not significant according to the results of the Cochrane’s *Q* test; otherwise, a random effect model was applied (Ma et al., 2018). The primary outcome of the study was the difference of changes of HbA1c between patients treated with DPP4 inhibitors and AGIs. Predefined subgroup analyses were performed to evaluate the potential influence of study characteristics including blinding, number of patients, baseline HbA1c, with or without background OADs, follow-up durations, and individual drugs of DPP4 inhibitor or AGI applied on the primary outcome. Medians of the continuous variables were used as cutoff value for stratification in subgroup analyses (Moher et al., 1998). The potential publication bias for the meta-analysis of each outcome was evaluated by the visual inspection of the symmetry of the funnel plots, as well as the Egger’s regression test (Egger et al., 1997). A *P* value < 0.05 indicates statistical significance.

## Results

### Database Search Result

**Figure 1:**
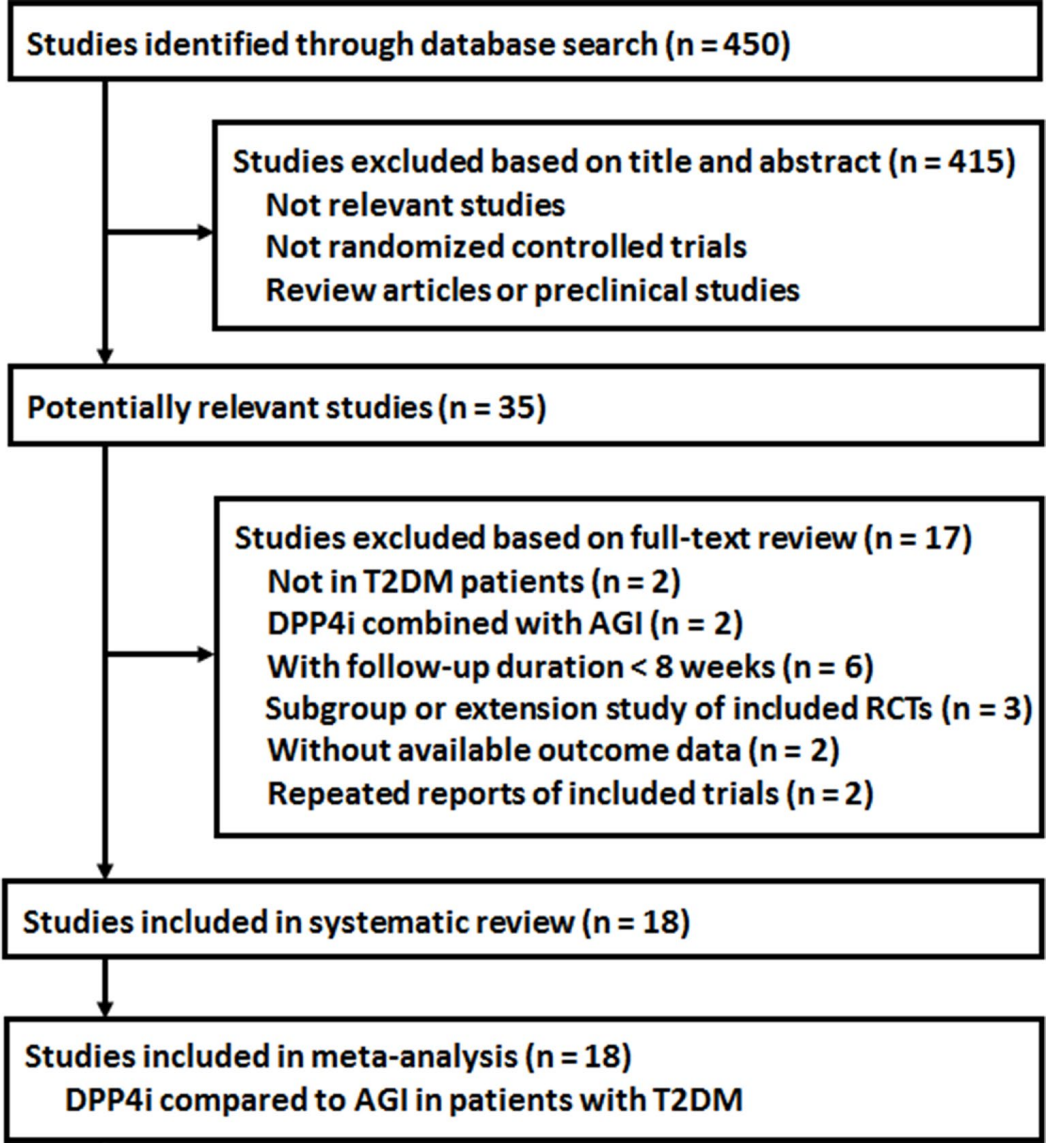
Flowchart of database search and study identification of the meta-analysis.

The process of database search was summarized in **Figure 1**. Briefly, 450 studies were obtained *via* the initial database search, and 415 studies were excluded based on analyses of titles and abstracts, mostly because these studies were irrelevant to current study objective. Of the remaining 35 studies that underwent full-text review, 17 studies were excluded because two of them did not include T2DM patients, two evaluated a combined effect of DPP4 inhibitors and AGIs, six were with follow-up durations <8 weeks, three were subgroup or extension studies of included RCTs, two were not with available outcome data, and another two were repeated reports of the included RCTs. Finally, 18 RCTs were included (Pan et al., 2008; Iwamoto et al., 2010a; Iwamoto et al., 2010b; Seino et al., 2011; Kawamori et al., 2012; Okada et al., 2013; Kobayashi et al., 2014; Mikada et al., 2014; Nakamura et al., 2014; Oe et al., 2015; Wang et al., 2015; Yokoh et al., 2015; Fujitani et al., 2016; Matsushima et al., 2016; Mori et al., 2016; Du et al., 2017; Koyama et al., 2018; Parthan et al., 2018).

### Study Characteristics and Quality Evaluation

Overall, our meta-analysis included 18 RCTs (Pan et al., 2008; Iwamoto et al., 2010a; Iwamoto et al., 2010b; Seino et al., 2011; Kawamori et al., 2012; Okada et al., 2013; Kobayashi et al., 2014; Mikada et al., 2014; Nakamura et al., 2014; Oe et al., 2015; Wang et al., 2015; Yokoh et al., 2015; Fujitani et al., 2016; Matsushima et al., 2016; Mori et al., 2016; Du et al., 2017; Koyama et al., 2018; Parthan et al., 2018) with a total of 4,051 T2DM patients. Since two studies included more than one intervention groups with different dosages of DPP4 inhibitors (Seino et al., 2011; Kawamori et al., 2012), we split the control group of AGI equally and included these datasets with different dosages of DPP4 inhibitors as independent comparisons into the meta-analysis, as indicated by the Cochrane’s Handbook guideline (Higgins and Green, 2011). Therefore, 22 comparisons of the efficacy between DPP4 inhibitors and AGIs in T2DM patients were included in our meta-analysis. The characteristics of included RCTs were summarized in **Table 1**. All of the included RCTs were published after 2008, and included T2DM patients from Japan and China except for one study which also included a small proportion of patients (<10%) from Romania and Spain (Pan et al., 2008). The sample sizes of the included studies varied from 16 to 661, with the mean ages varying from 49.7 to 97.5 years, and proportions of males ranging between 38.3 and 78.5%. The mean baseline HbA1c was 6.0∼8.6%, and the mean BMI was 21.5∼29.2 kg/m^2^. Nine RCTs included patients of initial therapy without background OADs (Pan et al., 2008; Iwamoto et al., 2010a; Iwamoto et al., 2010b; Seino et al., 2011; Kawamori et al., 2012; Mori et al., 2016; Du et al., 2017; Koyama et al., 2018; Parthan et al., 2018), while the other 13 RCTs compared the efficacy between DPP4 inhibitors and AGIs on the basis of background OADs (Okada et al., 2013; Kobayashi et al., 2014; Mikada et al., 2014; Nakamura et al., 2014; Oe et al., 2015; Wang et al., 2015; Yokoh et al., 2015; Fujitani et al., 2016; Matsushima et al., 2016). Five DPP4 inhibitors (alogliptin, linagliptin, saxagliptin, sitagliptin, and vildagliptin) and three AGIs (acarbose, miglitol, and voglibose) were applied respectively in the included RCTs, with the regularly recommended doses. The follow-up durations varied from 10 to 52 weeks. The details of quality evaluation for the included RCTs were summarized in **Table 2**. Overall, the quality of the included RCTs was moderate. Briefly, six of the included studies were double-blinded RCTs (Pan et al., 2008; Iwamoto et al., 2010a; Iwamoto et al., 2010b; Seino et al., 2011; Kawamori et al., 2012; Parthan et al., 2018), while the other 16 were open label studies (Okada et al., 2013; Kobayashi et al., 2014; Mikada et al., 2014; Nakamura et al., 2014; Oe et al., 2015; Wang et al., 2015; Yokoh et al., 2015; Fujitani et al., 2016; Matsushima et al., 2016; Mori et al., 2016; Du et al., 2017; Koyama et al., 2018). Six studies reported the methods of random sequence generation (Iwamoto et al., 2010b; Kobayashi et al., 2014; Oe et al., 2015; Yokoh et al., 2015; Matsushima et al., 2016; Mori et al., 2016). However, none of them reported the details of allocation concealment.

**Table 1:** Characteristics of the included studies.

Study	Country	Design	Sample size	Mean age	Male	HbA1c	BMI	Background OADs	Dpp4 inhibitors	AGI	Follow-up
				Years	%	%	kg/m^2^				Weeks
Pan et al. (2008)	China, Romania, and Spain	R, DB	661	51.9	61.2	8.6	26.3	None	Vildagliptin 50 mg bid	Acarbose 50∼100 mg tid	24
Iwamoto et al. (2010a)	Japan	R, DB	319	60.7	66.5	7.8	24.6	None	Sitagliptin 50 mg qd	Voglibose 0.2 mg tid	12
Iwamoto et al. (2010b)	Japan	R, DB	380	59.1	66.1	7.6	24.9	None	Vildagliptin 50 mg bid	Voglibose 0.2 mg tid	12
Seino et al., (2011) ^1^	Japan	R, DB	480	58.9	71.9	7.9	24.7	None	Alogliptin 6.25, 12.5, 25, and 50 mg qd	Voglibose 0.2 mg tid	52
Kawamori et al. (2012) ^2^	Japan	R, DB	481	59.8	70.4	8	25.1	None	Linagliptin 5 mg, 10 mg qd	Voglibose 0.2 mg tid	26
Okada et al. (2013)	Japan	R	35	65.6	38.3	7.8	24.4	Sulfonylurea	Sitagliptin 50 mg qd	Miglitol 50 mg tid	10
Nakamura et al. (2014)	Japan	R	55	67.5	50.9	7	26.6	Sulfonylurea, metformin, or pioglitazone	Sitagliptin 50 mg qd	Voglibose 0.2 mg tid	12
Kobayashi et al. (2014)	Japan	R	114	64.2	61.5	7.6	24.3	Sulfonylurea	Sitagliptin 50 mg qd	Voglibose 0.2 mg tid or miglitol 50 mg tid	24
Mikada et al. (2014)	Japan	R	28	59	78.5	7.2	29.2	Metformin or sulfonylurea	Sitagliptin 50 mg qd	Miglitol 50 mg tid	24
Oe et al. (2015)	Japan	R	80	67.2	57.5	7	26.7	Sulfonylurea, metformin, or pioglitazone	Sitagliptin 50 mg qd	Voglibose 0.2 mg tid	24
Wang et al. (2015)	China	R	81	64.6	46.3	8.3	NA	Metformin	Saxagliptin 5 mg qd	Acarbose 50 mg tid	52
Yokoh et al. (2015)	Japan	R	119	58.5	63.8	7.6	26.1	Metformin or pioglitazone	Sitagliptin 50 mg qd	Voglibose 0.2 mg tid or miglitol 50 mg tid	24
Mori et al. (2016)	Japan	R	78	67.2	78.2	6.6	21.5	None	Linagliptin 5 mg qd	Voglibose 0.2 mg tid	12
Matsushima et al. (2016)	Japan	R	241	63.2	59.3	7.9	25	Sulfonylurea, metformin, or pioglitazone	Sitagliptin 50 mg qd	Voglibose 0.2 mg tid	12
Fujitani et al. (2016)	Japan	R	382	61	55.1	7	25.4	None	Linagliptin 5 mg qd	Voglibose 0.2 mg tid	12
Du et al. (2017)	China	R	481	55.6	59.3	8.2	26.3	Metformin	Saxagliptin 5 mg qd	Acarbose 50∼100 mg tid	24
Parthan et al. (2018)	Japan	R, DB	20	49.7	60	6.9	25.7	None	Linagliptin 5 mg qd	Voglibose 0.2 mg tid	24
Koyama et al. (2018)	Japan	R	16	NA	NA	6	NA	None	Linagliptin 5 mg qd	Voglibose 0.3 mg tid	12

^1^The study by Seino et al. (2011) included four groups of alogliptin treatment with different dosages, and these were included as four comparisons.

^2^The study by Kawamori et al. (2012) included two groups of linagliptin treatment with different dosages, and these were included as four comparisons.

BMI, body mass index; OAD, oral antidiabetic drugs; DPP4, dipeptidyl peptidase-4; AGI, alpha-glucosidase inhibitors; R, randomized; DB, double blinded; NA, not available; qd, once daily; bid, twice daily; tid, three times daily.

**Table 2:** Quality evaluation of the included studies *via* Cochrane’s risk of bias tool.

	Random sequence generation	Allocation concealment	Blinding in performance	Blinding in outcome detection	Incomplete outcome data	Reporting bias	Other bias
Pan et al. (2008)	Unclear	Unclear	Low	Low	Low	Low	Unclear
Iwamoto et al. (2010a)	Low	Unclear	Low	Low	Low	Low	Low
Iwamoto et al. (2010b)	Unclear	Unclear	Low	Low	Low	Low	Low
Seino et al. (2011)	Unclear	Unclear	Low	Low	Low	Low	Low
Kawamori et al. (2012)	Unclear	Unclear	Low	Low	Low	Low	Low
Okada et al. (2013)	Unclear	Unclear	High	High	Low	Low	Low
Nakamura et al. (2014)	Unclear	Unclear	High	High	Low	Low	Low
Kobayashi et al. (2014)	Low	Unclear	High	High	Low	Low	Low
Mikada et al. (2014)	Unclear	Unclear	High	High	Low	Low	Low
Oe et al. (2015)	Low	Unclear	High	High	Low	Low	Low
Wang et al. (2015)	Unclear	Unclear	High	High	Low	Low	Low
Yokoh et al. (2015)	Low	Unclear	High	High	Low	Low	Low
Mori et al. (2016)	Low	Unclear	High	High	Low	Low	Low
Matsushima et al. (2016)	Low	Unclear	High	High	Low	Low	Low
Fujitani et al. (2016)	Unclear	Unclear	High	High	Low	Low	Low
Du et al. (2017)	Unclear	Unclear	High	High	Low	Low	Low
Parthan et al. (2018)	Unclear	Unclear	Low	Low	Low	Low	Unclear
Koyama et al. (2018)	Unclear	Unclear	High	High	Low	Low	Unclear

### Comparison Between DPP4 Inhibitors and AGIs on HbA1c in T2DM

**Figure 2:**
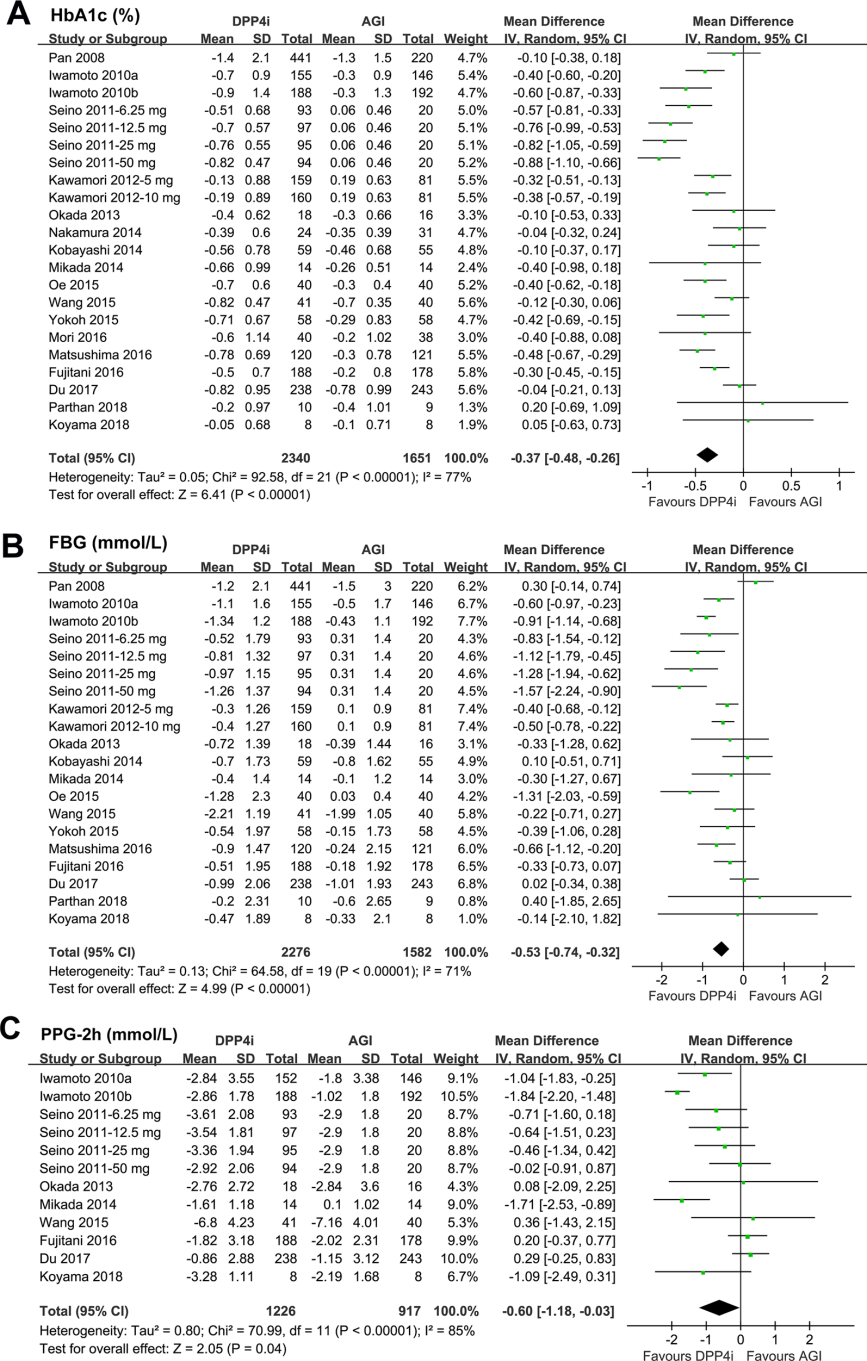
Forest plots for the meta-analysis of the hypoglycemic efficacy between dipeptidyl peptidase-4 (DPP4) inhibitors and α-glucosidase inhibitors (AGIs) in type 2 diabetes mellitus (T2DM) patients: **(A)** effect of DPP4 inhibitors and AGIs on glycosylated hemoglobin (HbA1c); **(B)** effect of DPP4 inhibitors and AGIs on FBG; and **(C)** effect of DPP4 inhibitors and AGIs on PPG-2h.

All of the 22 comparisons reported the efficacy of DPP4 inhibitors *versus* AGIs on HbA1c changes in T2DM patients. Significant heterogeneity was detected among the included RCTs (*P* for Cochrane’s *Q* test < 0.001, *I* ^2^ = 77%). Pooled results with a random effect model showed that treatment with DPP4 inhibitors was associated with a significant lower reduction of HbA1c as compared with AGIs (WMD: −0.37%, 95% CI: −0.48 to −0.26, *p* < 0.001; **Figure 2A**). Subsequent subgroup analyses indicated a significant lower reduction of HbA1c in patients treated with DPP4 inhibitors than AGIs, which was independent of the study design, patient number of the included RCTs, HbA1c level at baseline, with or without background OADs, and follow-up durations (*P* for efficacies all < 0.05; **Table 3**). Moreover, the results of stratified analyses indicated that the reduction of HbA1c by DPP4 inhibitors compared with AGIs was more remarkable in double-blinded studies than open-label trials (*P* for subgroup difference = 0.005), and in patients without background OADs than those with background OADs (*P* for subgroup difference = 0.01). In addition, a trend of lower reduction of HbA1c was observed in studies with longer follow-up duration (52 weeks) than those with shorter follow-up duration (*P* for subgroup difference = 0.07). Stratified analyses according to the specific medications of DPP4 inhibitors and AGIs used in the RCTs showed that the benefits of DPP4 inhibitors *versus* AGIs on HbA1c was significant in studies with alogliptin, linagliptin, and sitagliptin, but not significant in studies with saxagliptin or vildagliptin, while significant in studies with voglibose, but not significant in studies with acarbose or miglitol.

**Table 3:** Subgroup analysis for the effects of DPP4i compared with AGI on HbA1c.

Variables	Datasets (patients)	WMD (95% CI)	*P* for subgroup effect	*I*^2^	*P* for subgroup difference
**Study design**					
R, DB	10 (2,301)	−0.52 [−0.68, −0.35]	<0.001	77%	
R	12 (1,690)	−0.24 [−0.35, −0.13]	<0.001	51%	0.005
**Sample size**					
>100	16 (3,689)	−0.40 [−0.53, −0.27]	<0.001	82%	
≤100	6 (302)	−0.24 [−0.40, −0.08]	0.003	17%	0.12
**Baseline HbA1c (%)**					
>7	15 (3,349)	−0.41 [−0.55, −0.27]	<0.001	83%	
≤7	7 (642)	−0.27 [−0.39, −0.15]	<0.001	7%	0.14
**Background OADs**					
Yes	9 (1,230)	−0.23 [−0.36, −0.09]	<0.001	61%	
No	13 (2,761)	−0.47 [−0.62, −0.33]	<0.001	75%	0.01
**Follow-up duration (weeks)**					
≤12	8 (1,471)	−0.34 [−0.47, −0.21]	<0.001	47%	
24∼26	9 (1,980)	−0.25 [−0.37, −0.13]	<0.001	45%	
52	5 (540)	−0.63 [−0.93, −0.32]	<0.001	90%	0.07
**DPP4i medications**					
Alogliptin	4 (459)	−0.76 [−0.89, −0.64]	<0.001	19%	
Linagliptin	6 (960)	−0.32 [−0.42, −0.22]	<0.001	0%	
Saxagliptin	2 (562)	−0.08 [−0.20, 0.05]	0.22	0%	
Sitagliptin	8 (969)	−0.31 [−0.44, −0.19]	<0.001	41%	
Vildagliptin	2 (1,041)	−0.35 [−0.84, 0.14]	0.16	84%	<0.001
**AGI medications**					
Acarbose	3 (1,223)	−0.08 [−0.20, 0.03]	0.16	0%	
Miglitol	2 (62)	−0.21 [−0.55, 0.14]	0.24	0%	
Voglibose	15 (2,476)	−0.47 [−0.59, −0.34]	<0.001	73%	<0.001

WMD, weighed mean difference; CI, confidence interval; R, randomized; DB, double blinded; DPP4i, dipeptidyl peptidase-4 inhibitors; AGI, alpha-glucosidase inhibitors.

### Comparison Between DPP4 Inhibitors and AGIs on FBG and PPG-2h in T2DM

Pooled results of 20 comparisons showed that treatment with DPP4 inhibitors was associated with a significantly lower reduction of FBG as compared with AGIs (WMD: −0.53 mmol/L, 95% CI: −0.74 to −0.32, *P* < 0.001; **Figure 2B**) with significant heterogeneity (*P* for Cochrane’s *Q* test < 0.001; *I* ^2^ = 71%). Moreover, we found that DPP4 inhibitors were associated with a significantly lower reduction of PPG-2h as compared with AGIs in T2DM patients (WMD: −0.60 mmol/L, 95% CI: −1.18 to −0.03, *P* = 0.04; **Figure 2C**) with significant heterogeneity (*P* for Cochrane’s *Q* test < 0.001; *I* ^2^ = 85%).

### Comparison Between DPP4 Inhibitors and AGIs on Body Weight, Symptomatic Hypoglycemia, and Risk of GIAEs

**Figure 3:**
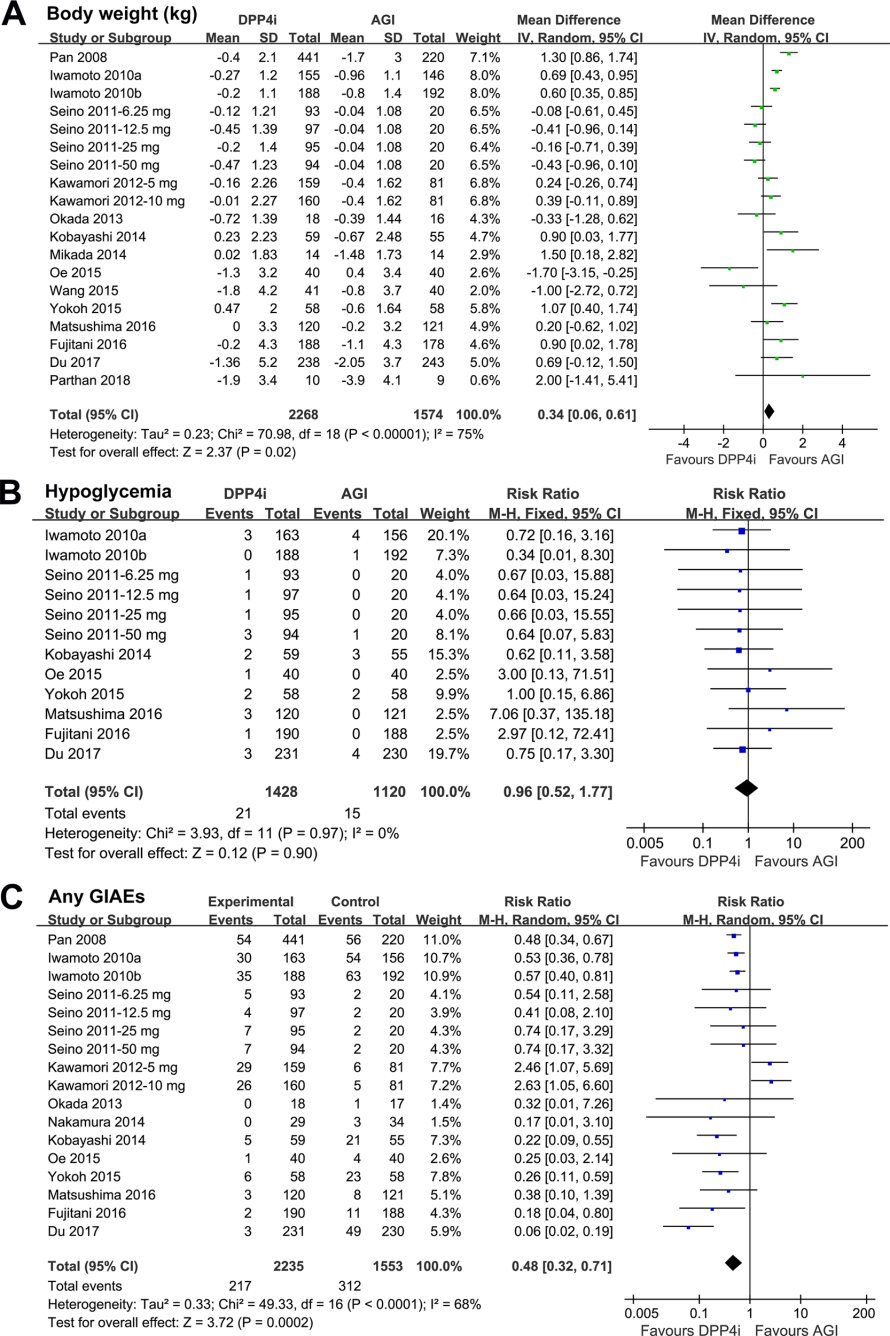
Forest plots for the meta-analysis of the safety outcomes between DPP4 inhibitors and AGIs in T2DM patients: **(A)** effect of DPP4 inhibitors and AGIs on body weight; **(B)** effect of DPP4 inhibitors and AGIs on the incidence of symptomatic hypoglycemia; and **(C)** effect of DPP4 inhibitors and AGIs on the incidence of any gastrointestinal adverse events (GIAEs).

Meta-analysis with 19 comparisons showed that DPP4 inhibitors significantly increased body weight as compared with AGIs in T2DM patients (WMD: 0.34 kg, 95% CI: 0.06 to 0.61, *P* = 0.02; **Figure 3A**) with significant heterogeneity (*P* for Cochrane’s *Q* test < 0.001; *I* ^2^ = 75%). We also found that DPP4 inhibitors did not significantly affect the risk of symptomatic hypoglycemia as compared with AGIs (RR: 0.96, 95% CI: 0.52 to 1.77, *P* = 0.90; **Figure 3B**) without significant heterogeneity (*P* for Cochrane’s *Q* test = 0.97; *I* ^2^ = 0%), while treatment with DPP4 inhibitors was associated with significantly reduced risk of any GIAEs as compared with AGIs (RR: 0.48, 95% CI: 0.32 to 0.71, *P* < 0.001; **Figure 3C**) with significant heterogeneity (*P* for Cochrane’s *Q* test < 0.001; *I* ^2^ = 68%).

### Publication Bias

**Figure 4:**
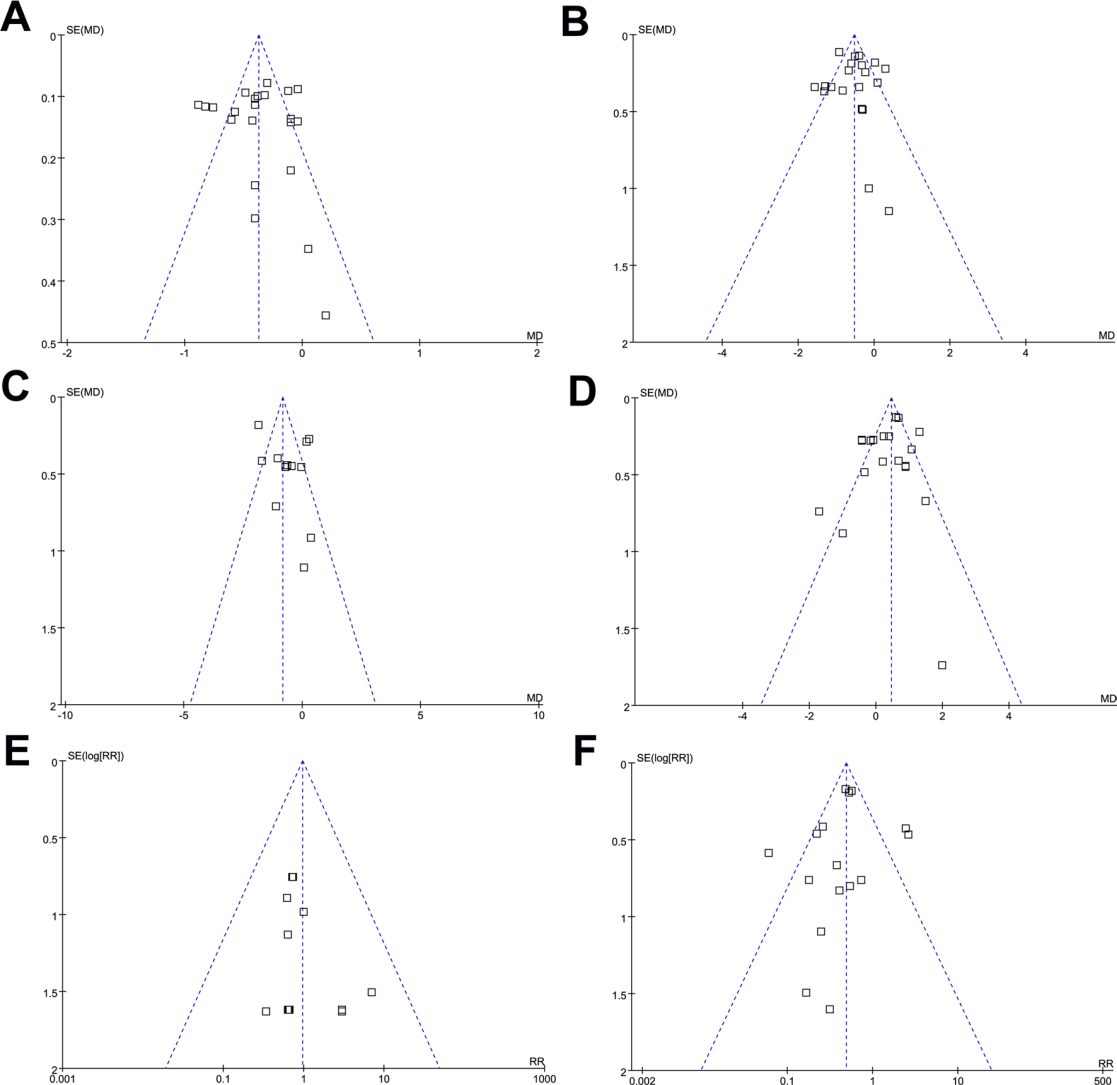
Funnel plots for the meta-analyses comparing the efficacy and safety outcomes between DPP4 inhibitors and AGIs in T2DM patients; **(A)** HbA1c; **(B)** FBG; **(C)** PPG-2h; **(D)** body weight; **(E)** symptomatic hypoglycemia; and **(F)** any GIAEs.

Funnel plots for meta-analyses of the effects on changes of HbA1c, FBG, PPG-2h, and body weight, and the risks of symptomatic hypoglycemia and any GIAEs, were presented in **Figure 4A–F**, which were symmetric on visual inspection, indicating no significant publication biases. Results of Egger’s regression tests also demonstrated no significance in publication biases (*P* for Egger’s regression tests = 0.38, 0.52, 0.61, 0.58, 0.48, and 0.21 respectively).

## Discussion

In this updated meta-analysis of 18 RCTs with 4,051 T2DM patients, we found that DPP4 inhibitors confer better glycemic control than AGIs, as evidenced by significantly reduced HbA1c, FBG, and PPG-2h by DPP4 inhibitors compared to AGIs. Moreover, treatment with DPP4 inhibitors is associated with much less GIAEs and unaffected hypoglycemia, but a modest increased body weight as compared with AGIs. These results indicate that for T2DM patients, DPP4 inhibitors are superior to AGIs for better glycemic control but lower risks of GIAEs.

A previous meta-analysis (Cai et al., 2015) including nine RCTs showed that treatment with DPP4 inhibitors was associated with a lower reduction of HbA1c and a less incidence of gastrointestinal discomfort as compared with AGIs, while the other meta-analysis (Gao et al., 2018) including 11 RCTs published up to 2016 showed that AGI treatment was associated with a significantly lower reduction in HbA1c than DPP4 inhibitors. Many related RCTs have been published since the previous meta-analyses (Oe et al., 2015; Fujitani et al., 2016; Matsushima et al., 2016; Mori et al., 2016; Du et al., 2017; Koyama et al., 2018; Parthan et al., 2018), which were included in this updated meta-analysis. Our meta-analysis has a few strengths as compared with the previous meta-analysis of the similar topic (Cai et al., 2015). Firstly, our updated meta-analysis included 18 RCTs of 4,051 T2DM patients, which is much larger than those of the previous ones, which enables us to perform subgroup analyses to confirm the robustness of our findings regarding the superiority of DPP4 inhibitors on glycemic control over AGIs. Results of the subgroup analysis confirmed that treatment with the DPP4 inhibitors was associated with significantly reduced HbA1c as compared with AGIs. The superiority of glycemic control of DPP4 inhibitors over AGIs was independent of study design, patient number of the included RCTs, HbA1c level at baseline, with or without background OADs, and follow-up durations, indicating the stability of the findings. Secondly, results of our subgroup analysis also suggested some interesting findings. For example, we found that the reduction of HbA1c by DPP4 inhibitors compared with AGIs was more remarkable in double-blinded studies than open-label trials. Since open-label trials are vulnerable to various bias, these results indicated that the actual hypoglycemic efficacy of DPP4 inhibitors compared with AGIs may be stronger than the overall results (−0.30% in previous meta-analysis and−0.37% in our study). In addition, we found a trend of lower reduction of HbA1c by DPP4 inhibitors compared with AGIs in studies with longer follow-up duration (52 weeks) than those with shorter follow-up duration. These results suggest the optimal durability of the hypoglycemic efficacy of DPP4 inhibitors. Thirdly, we found that despite of significant decreased FBG by DPP4 inhibitors compared with AGIs, treatment with DPP4 inhibitors also significantly decreased PPG as compared with AGIs in T2DM patients, which is not reported in previous meta-analysis (Cai et al., 2015). This is of clinical significance since AGIs are often considered as the OADs of choice to lower PPG because of its direct pharmacological action, which is to inhibit the absorption of carbohydrates during the meals (Liu and Ma, 2017; Shimabukuro et al., 2017). Based on our results, DPP4 inhibitors should be considered in patients with high PPG levels. Moreover, meal intake–related glycemic excursion has been recognized as an important contributor to glycemic variability, which has been validated as another risk factor for morbidity and mortality in T2DM patients independent of glycemic control (Gorst et al., 2015; Nusca et al., 2018). Therefore, our finding indicated that DPP4 inhibitors may be an alternative category of OADs to stabilize glycemic variability, although studies are needed to confirm our findings.

The potential mechanisms underlying the potential superiority of DPP4 inhibitors on glycemic control to AGIs may be primarily explained by the differences of the pharmacological mechanisms of the two categories of OADs. As mentioned previously, the AGIs mainly lower the PPG *via* inhibiting the absorption of carbohydrates during the meal (Liu and Ma, 2017). However, DPP4 inhibitors could exert both the fasting and postprandial hypoglycemic efficacies *via* reducing glucagon secretion during fasting and stimulating the glucose-dependent insulin secretion during the meal (Chen et al., 2015). Besides, DPP4 inhibitors may also be associated with better patient compliance, since most DPP4 inhibitors are to be taken once daily, while all of the AGIs are to be taken three times daily. This could be an alternative mechanism underlying the better glycemic control as observed in patients taking DPP4 inhibitors compared to those taking AGIs.

Our results confirmed the findings of the previous studies that treatment with DPP4 inhibitors in T2DM patients is associated with significantly decreased risk of GIAEs and unaffected hypoglycemia, but a moderate increased body weight as compared with AGIs (Cai et al., 2015; Wu et al., 2017). Because high incidence of GIAEs is a common reason for the discontinuation of AGIs in T2DM patients, based on our findings, DPP4 inhibitors should be recommended to patients who are unable to tolerate AGIs as an initial therapy for T2DM or in combination with other OADs. The mechanisms underlying the unaffected risk of hypoglycemia in patients taking DPP4 inhibitors as compared with those taking AGIs may rely on the glucose-dependent insulin stimulatory effect of DPP4 inhibitors (Flock et al., 2007; Vardarli et al., 2011). Moreover, the modest effect of body weight gain in DPP4 inhibitors as compared with AGIs could be explained by the increased GIP level after treatment of DPP4 inhibitors. The GIP has been recognized as a peptide which could enhance the uptake of lipids into the adipocytes in the overnutrition condition (Thondam et al., 2017). However, from our point of view, the gain of body weight after treatment with DPP4 inhibitors as compared with AGIs is moderate (0.34 kg), which does not outweigh the potential benefits of DPP4 inhibitors on glycemic control and GIAE risk.

Our study has limitations. Firstly, significant heterogeneities exist for meta-analysis comparing the efficacies of DPP4 inhibitors and AGIs on HbA1c, FBG, PPG-2h, body weight, and incidence of GIAEs. The differences of study characteristics, such as the baseline HbA1c, T2DM durations, comorbidities, and concurrent medications including OADs, may contribute to the heterogeneities. While the influences of these factors could not be determined since the stratified outcomes according to the above characteristics were not reported in the included RCTs. Moreover, we performed the meta-analysis on the study level instead of on the individual patient level because we did not have access to the individual patient data. This prevented us from performing intensified analyses of the heterogeneity. In addition, as compared with AGIs, the influences of DPP4 inhibitors on clinical outcomes, such as incidences of cardiovascular events and overall mortality, remained to be determined. Finally, the findings of the meta-analysis were almost all based on the RCTs in Asian patients. Since carbohydrates account for a larger proportion of diet structures of the Asians, AGIs are often prescribed in Asian T2DM patients (Zhu et al., 2013). Whether DPP4 inhibitors are superior to AGIs in glycemic control in other ethnic group should be investigated in the future.

## Conclusions

In conclusion, our meta-analysis showed that DPP4 inhibitors are superior to AGIs in T2DM patients for better glycemic control but lower risks of GIAEs.

## Author Contributions

ZL and LZ carried out the acquisition and analysis of data, and drafting the manuscript. JY performed the drafting and revising of the manuscript. LY participated in the design and helped to revise the manuscript. All authors have read and approved the final version of the manuscript prior to submission.

## Conflict of Interest Statement

The authors declare that the research was conducted in the absence of any commercial or financial relationships that could be construed as a potential conflict of interest.

## References

[B1] Cai C. (2019). Interpretation of the management requirements of national guidelines for the prevention and control of diabetes in primary care (2018). Zhonghua Nei Ke Za Zhi 58, 147–149. 10.3760/cma.j.issn.0578-1426.2019.02.013 30704203

[B2] Cai X. Yang W. Zhou L. Zhang S. Han X. Ji L. (2015). Comparisons of the efficacy of glucose control, lipid profile, and beta-cell function between DPP-4 inhibitors and AGI treatment in type 2 diabetes patients: a meta-analysis. Endocrine 50, 590–597. 10.1007/s12020-015-0653-3 26048437

[B3] Chen X. W. He Z. X. Zhou Z. W. Yang T. Zhang X. Yang Y. X. (2015). Clinical pharmacology of dipeptidyl peptidase 4 inhibitors indicated for the treatment of type 2 diabetes mellitus. Clin. Exp. Pharmacol. Physiol. 42, 999–1024. 10.1111/1440-1681.12455 26173919

[B4] Chu W. M. Ho H. E. Huang K. H. Tsan Y. T. Liou Y. S. Wang Y. H. (2017). The prescribing trend of oral antidiabetic agents for type 2 diabetes in Taiwan: an 8-year population-based study. Medicine (Baltimore) 96, e8257. 10.1097/MD.0000000000008257 29068991 PMC5671824

[B5] Du J. Liang L. Fang H. Xu F. Li W. Shen L. (2017). Efficacy and safety of saxagliptin compared with acarbose in Chinese patients with type 2 diabetes mellitus uncontrolled on metformin monotherapy: results of a Phase IV open-label randomized controlled study (the SMART study). Diabetes Obes. Metab. 19, 1513–1520. 10.1111/dom.12942 28296055

[B6] Egger M. Davey Smith G. Schneider M. Minder C. (1997). Bias in meta-analysis detected by a simple, graphical test. BMJ 315, 629–634. 10.1136/bmj.315.7109.629 9310563 PMC2127453

[B7] Flock G. Baggio L. L. Longuet C. Drucker D. J. (2007). Incretin receptors for glucagon-like peptide 1 and glucose-dependent insulinotropic polypeptide are essential for the sustained metabolic actions of vildagliptin in mice. Diabetes 56, 3006–3013. 10.2337/db07-0697 17717280

[B8] Fujitani Y. Fujimoto S. Takahashi K. Satoh H. Hirose T. Hiyoshi T. (2016). Effects of linagliptin monotherapy compared with voglibose on postprandial blood glucose responses in Japanese patients with type 2 diabetes: Linagliptin Study of Effects on Postprandial blood glucose (L-STEP). Diabetes Res. Clin. Pract. 121, 146–156. 10.1016/j.diabres.2016.09.014 27710821

[B9] Gao X. Cai X. Yang W. Chen Y. Han X. Ji L. (2018). Meta-analysis and critical review on the efficacy and safety of alpha-glucosidase inhibitors in Asian and non-Asian populations. J. Diabetes Investig. 9, 321–331. 10.1111/jdi.12711 PMC5835463 28685995

[B10] Gorst C. Kwok C. S. Aslam S. Buchan I. Kontopantelis E. Myint P. K. (2015). Long-term glycemic variability and risk of adverse outcomes: a systematic review and meta-analysis. Diabetes Care 38, 2354–2369. 10.2337/dc15-1188 26604281

[B11] Higgins J. Green S. (2011). “Cochrane Handbook for Systematic Reviews of Interventions Version 5.1.0,” in The Cochrane Collaboration. London, UK: Wiley www.cochranehandbook.org.

[B12] Higgins J. P. Thompson S. G. Deeks J. J. Altman D. G. (2003). Measuring inconsistency in meta-analyses. BMJ 327, 557–560. 10.1136/bmj.327.7414.557 12958120 PMC192859

[B13] Iwamoto Y. Kashiwagi A. Yamada N. Terao S. Mimori N. Suzuki M. (2010a). Efficacy and safety of vildagliptin and voglibose in Japanese patients with type 2 diabetes: a 12-week, randomized, double-blind, active-controlled study. Diabetes Obes. Metab. 12, 700–708. 10.1111/j.1463-1326.2010.01222.x 20590747 PMC2916214

[B14] Iwamoto Y. Tajima N. Kadowaki T. Nonaka K. Taniguchi T. Nishii M. (2010b). Efficacy and safety of sitagliptin monotherapy compared with voglibose in Japanese patients with type 2 diabetes: a randomized, double-blind trial. Diabetes Obes. Metab. 12, 613–622. 10.1111/j.1463-1326.2010.01197.x 20590736

[B15] Jeon J. Y. Lee S. J. Lee S. Kim S. J. Han S. J. Kim H. J. (2018). Failure of monotherapy in clinical practice in patients with type 2 diabetes: the Korean National Diabetes Program. J. Diabetes Investig. 9, 1144–1152. 10.1111/jdi.12801 PMC6123024 29328551

[B16] Kawamori R. Inagaki N. Araki E. Watada H. Hayashi N. Horie Y. (2012). Linagliptin monotherapy provides superior glycaemic control versus placebo or voglibose with comparable safety in Japanese patients with type 2 diabetes: a randomized, placebo and active comparator-controlled, double-blind study. Diabetes Obes. Metab. 14, 348–357. 10.1111/j.1463-1326.2011.01545.x 22145698

[B17] Kobayashi K. Yokoh H. Sato Y. Takemoto M. Uchida D. Kanatsuka A. (2014). Efficacy and safety of the dipeptidyl peptidase-4 inhibitor sitagliptin compared with alpha-glucosidase inhibitor in Japanese patients with type 2 diabetes inadequately controlled on sulfonylurea alone (SUCCESS-2): a multicenter, randomized, open-label, non-inferiority trial. Diabetes Obes. Metab. 16, 761–765. 10.1111/dom.12264 24447683

[B18] Koyama T. Tanaka A. Yoshida H. Oyama J. I. Toyoda S. Sakuma M. (2018). Comparison of the effects of linagliptin and voglibose on endothelial function in patients with type 2 diabetes and coronary artery disease: a prospective, randomized, pilot study (EFFORT). Heart Vessels 33, 958–964. 10.1007/s00380-018-1136-2 29427024

[B19] Liu Z. Ma S. (2017). Recent advances in synthetic alpha-Glucosidase inhibitors. ChemMedChem 12, 819–829. 10.1002/cmdc.201700216 28498640

[B20] Ma X. Lin L. Qu Z. Zhu M. Chu H. (2018). Performance of between-study heterogeneity measures in the Cochrane Library. Epidemiology 29, 821–824. 10.1097/EDE.0000000000000857 29847495 PMC6167168

[B21] Matsushima Y. Takeshita Y. Kita Y. Otoda T. Kato K. Toyama-Wakakuri H. (2016). Pleiotropic effects of sitagliptin versus voglibose in patients with type 2 diabetes inadequately controlled via diet and/or a single oral antihyperglycemic agent: a multicenter, randomized trial. BMJ Open Diabetes Res Care 4, e000190. 10.1136/bmjdrc-2015-000190 PMC4838664 27110370

[B22] Mikada A. Narita T. Yokoyama H. Yamashita R. Horikawa Y. Tsukiyama K. (2014). Effects of miglitol, sitagliptin, and initial combination therapy with both on plasma incretin responses to a mixed meal and visceral fat in over-weight Japanese patients with type 2 diabetes. Diabetes Res. Clin. Pract. 106, 538–547. 10.1016/j.diabres.2014.09.040 25451890

[B23] Moher D. Liberati A. Tetzlaff J. Altman D. G. (2009). Preferred reporting items for systematic reviews and meta-analyses: the PRISMA statement. BMJ 339, b2535. 10.1136/bmj.b2535 19622551 PMC2714657

[B24] Moher D. Pham B. Jones A. Cook D. J. Jadad A. R. Moher M. (1998). Does quality of reports of randomised trials affect estimates of intervention efficacy reported in meta-analyses? Lancet 352, 609–613. 10.1016/S0140-6736(98)01085-X 9746022

[B25] Mori K. Emoto M. Shoji T. Inaba M. (2016). Linagliptin monotherapy compared with voglibose monotherapy in patients with type 2 diabetes undergoing hemodialysis: a 12-week randomized trial. BMJ Open Diabetes Res Care 4, e000265. 10.1136/bmjdrc-2016-000265 PMC4964246 27547421

[B26] Nakamura K. Oe H. Kihara H. Shimada K. Fukuda S. Watanabe K. (2014). DPP-4 inhibitor and alpha-glucosidase inhibitor equally improve endothelial function in patients with type 2 diabetes: EDGE study. Cardiovasc. Diabetol. 13, 110. 10.1186/s12933-014-0110-2 25074318 PMC4149239

[B27] Nusca A. Tuccinardi D. Albano M. Cavallaro C. Ricottini E. Manfrini S. (2018). Glycemic variability in the development of cardiovascular complications in diabetes. Diabetes Metab. Res. Rev. 34, e3047. 10.1002/dmrr.3047 30028067

[B28] Oe H. Nakamura K. Kihara H. Shimada K. Fukuda S. Takagi T. (2015). Comparison of effects of sitagliptin and voglibose on left ventricular diastolic dysfunction in patients with type 2 diabetes: results of the 3D trial. Cardiovasc. Diabetol. 14, 83. 10.1186/s12933-015-0242-z 26084668 PMC4473835

[B29] Okada K. Yagyu H. Kotani K. Yamazaki H. Ozaki K. Takahashi M. (2013). Effects of miglitol versus sitagliptin on postprandial glucose and lipoprotein metabolism in patients with type 2 diabetes mellitus. Endocr. J. 60, 913–922. 10.1507/endocrj.EJ13-0019 23574730

[B30] Pan C. Yang W. Barona J. P. Wang Y. Niggli M. Mohideen P. (2008). Comparison of vildagliptin and acarbose monotherapy in patients with Type 2 diabetes: a 24-week, double-blind, randomized trial. Diabet. Med. 25, 435–441. 10.1111/j.1464-5491.2008.02391.x 18341596

[B31] Parthan G. Bhansali S. Kurpad A. V. Walia R. Bhat K. Bhansali A. (2018). Effect of Linagliptin and Voglibose on metabolic profile in patients with Type 2 Diabetes: a randomized, double-blind, placebo-controlled trial. BMC Pharmacol. Toxicol. 19, 38. 10.1186/s40360-018-0228-z 29970184 PMC6030784

[B32] Seino Y. Fujita T. Hiroi S. Hirayama M. Kaku K. (2011). Efficacy and safety of alogliptin in Japanese patients with type 2 diabetes mellitus: a randomized, double-blind, dose-ranging comparison with placebo, followed by a long-term extension study. Curr. Med. Res. Opin. 27, 1781–1792. 10.1185/03007995.2011.599371 21806314

[B33] Shimabukuro M. Tanaka A. Sata M. Dai K. Shibata Y. Inoue Y. (2017). alpha-Glucosidase inhibitor miglitol attenuates glucose fluctuation, heart rate variability and sympathetic activity in patients with type 2 diabetes and acute coronary syndrome: a multicenter randomized controlled (MACS) study. Cardiovasc. Diabetol. 16, 86. 10.1186/s12933-017-0571-1 28683829 PMC5501494

[B34] Thondam S. K. Daousi C. Wilding J. P. Holst J. J. Ameen G. I. Yang C. (2017). Glucose-dependent insulinotropic polypeptide promotes lipid deposition in subcutaneous adipocytes in obese type 2 diabetes patients: a maladaptive response. Am. J. Physiol. Endocrinol. Metab. 312, E224–E233. 10.1152/ajpendo.00347.2016 28073779

[B35] Vardarli I. Nauck M. A. Kothe L. D. Deacon C. F. Holst J. J. Schweizer A. (2011). Inhibition of DPP-4 with vildagliptin improved insulin secretion in response to oral as well as “isoglycemic” intravenous glucose without numerically changing the incretin effect in patients with type 2 diabetes. J. Clin. Endocrinol. Metab. 96, 945–954. 10.1210/jc.2010-2178 21239518

[B36] Wang M. M. Lin S. Chen Y. M. Shu J. Lu H. Y. Zhang Y. J. (2015). Saxagliptin is similar in glycaemic variability more effective in metabolic control than acarbose in aged type 2 diabetes inadequately controlled with metformin. Diabetes Res. Clin. Pract. 108, e67–e70. 10.1016/j.diabres.2015.02.022 25841300

[B37] Weng J. (2016). Evolution in the Chinese Diabetes Society Standards of Care for Type 2 Diabetes. Diabetes Metab. Res. Rev. 32, 440–441. 10.1002/dmrr.2826 27464264

[B38] Wu S. Chai S. Yang J. Cai T. Xu Y. Yang Z. (2017). Gastrointestinal adverse events of Dipeptidyl Peptidase 4 Inhibitors in Type 2 Diabetes: a systematic review and network meta-analysis. Clin. Ther. 39, 1780–1789 e1733. 10.1016/j.clinthera.2017.07.036 28827024

[B39] Yokoh H. Kobayashi K. Sato Y. Takemoto M. Uchida D. Kanatsuka A. (2015). Efficacy and safety of the dipeptidyl peptidase-4 inhibitor sitagliptin compared with alpha-glucosidase inhibitor in Japanese patients with type 2 diabetes inadequately controlled on metformin or pioglitazone alone (Study for an Ultimate Combination Therapy to Control Diabetes with Sitagliptin-1): a multicenter, randomized, open-label, non-inferiority trial. J. Diabetes Investig. 6, 182–191. 10.1111/jdi.12282 PMC4364853 25802726

[B40] Zhu Q. Tong Y. Wu T. Li J. Tong N. (2013). Comparison of the hypoglycemic effect of acarbose monotherapy in patients with type 2 diabetes mellitus consuming an Eastern or Western diet: a systematic meta-analysis. Clin. Ther. 35, 880–899. 10.1016/j.clinthera.2013.03.020 23602502

